# Applying Context: African Praxis, Ubuntu Ethics, and an Applied Model

**DOI:** 10.1002/yd.70002

**Published:** 2025-08-15

**Authors:** Oliver Seale, Simon Kagwe

**Affiliations:** ^1^ Ali Mazrui Centre for Higher Education Studies University of Johannesburg Oliver Seale, Johannesburg South Africa; ^2^ United States International University Africa Simon Kagwe, Nairobi Kenya

## Abstract

In this article, the authors present and engage with the conceptual and theoretical underpinning of leadership education and leadership development in Africa. Specifically, leadership education programs which are focusing on nurturing a new generation of leaders, adept at navigating the complexities of a globalized world, while remaining deeply rooted in their national and local heritage. They locate African leadership theory and praxis in the context of change, and examine how the International Leadership Association (ILA) General Principles for Leadership Programs (2021) provides an organizing framework for the redesigned Executive MBA program at the African Leadership University in Kigali, Rwanda.

## 1 Introduction

Xatyiswa Maqashalala (2025), in her Doctoral dissertation, claimed African countries adopted leadership models from their former colonizers. She further posited that the colonial influence in Western leadership philosophies and frameworks creates a perceptual misalignment between the cultural framework of the affected people and the systems that govern them. This disjunction, suggested Maqashalala (2025), raises important questions about cultural authenticity, identity, and the preservation of Indigenous wisdom in the face of globalized ideals. Oelofsen and Abimbola (2018) concurred that applying Western philosophical ideas and frameworks to Africa may have some merit. However, the call to decolonize our thinking about Africa, in applying African solutions to its problems, is becoming more voluble.

The ILA General Principles for Leadership Programs (2021) foregrounds the leadership context in the form of political, economic, social, and environmental drivers as key for transformative leadership in Africa. More so, its implications for inclusive and collective leadership, on the Continent, in the spirit and philosophy of *Ubuntu*.

Ubuntu is a traditional African concept, which is sometimes translated as ‘I am because we are.ntu embodies all those virtues that maintain harmony and the spirit of sharing among the members of a society.

For Nzimakwe (2014), Ubuntu is grounded in a philosophy of collectivism and relationships over material things, including ownership of opportunities, responsibilities, and challenges. Its key characteristics the author says are: (i) the human experience of treating people with respect; (ii) humanness, which means that being human, comprises values such as universality and sharing and treating and respecting others as human beings; (iii) a way of life contributing positively to sustaining the wellbeing of people, the community or society; and (iv) a non‐racial philosophy applicable to all people. Leaders with an ubuntu philosophy focus on and model through their actions the importance of respecting the individual, value collaborative work in teams, and provide an enabling and supportive working environment (Nzimakwe 2014).

## 2 Leadership Context

In our contemporary world characterized by change and complexity, the preceding theoretical constructs and praxis of leadership seem no longer appropriate for addressing challenges and opportunities in the 21^st^‐century organization. A report titled *Global Human Capital Trends 2019*, commissioned by Deloitte, shows 80% of the respondents indicated that leadership in the 21^st^‐century contemporary organization has unique and new requirements that are important for their organization's success. Additional focus areas for leadership identified in this report were—inclusion, fairness, social responsibility, understanding the role of automation, and leading in a network. These mentioned requirements were not part of their organization's leadership manifesto a decade ago. What the report also reveals is that in these times of complexity and change, many organizations are not satisfied with their leadership development programs (Schwartz et al. 2019).

### 2.1 Traditional Approaches

Traditional leadership expectations and outcomes are still relevant in the contemporary organization (Swartz et al., 2019), but should be combined with a set of new competencies and capabilities given the contextual specificities of constant change and complexity in our world today. Competencies and capabilities, the authors continued, are not enough. Of equal importance for the organization is enabling culture, the appropriate structure, and the management practices to identify and cultivate these leaders. Contextualization, developing new leadership competencies, and embedding the right culture in an organization are all critical cogs of an effective leadership strategy (Schwartz et al. 2019).

Some of these traditional approaches embedded a philosophy that promulgated an individual westernized leadership paradigm, in the ‘trait theory’ tradition and ‘heroic’ sense, which inspired and influenced others to solve problems and achieve goals. Although it was originally proposed in the 1800s, trait theory is often criticized as lacking a scientific foundation, not being inclusive, and not considering the leadership context or environmental factors that often shape the personality and philosophy of those identified as great leaders. In the post‐modern, knowledge and technological era, however, this view of people as being powerless, with no vision or ability to change, led like sheep, is no longer appropriate and applicable, especially in the context of African leadership (Seale 2019).

Seale (2021) agrees, stating a new concept of ‘post‐heroic’ leadership is emerging, which is grounded in bottom‐up transformation, driven by distributed leadership, power‐sharing, and organizational coalitions. This notion resonates with commentators who have written on team leadership, distributed leadership, and participatory leadership.

The trait theory and the heroic leadership paradigm emphasized individual qualities and the notion of a single, charismatic leader who drives success. This approach can be seen as promoting a Westernized, individualistic perspective on leadership. The main criticism of the trait theory, suggested by Harrison (2018), is the absence of empirical research to corroborate it. In this perspective, the trait theory is not based on the scientific method, but it is based on personal characteristics developed through continuous professional training and development. Although trait leadership focuses on the inherent qualities of the leader, it may have cultural bias and ignore the contextual specificities of the leadership environment.

Different leadership styles apply to different situations (Sivaruban 2021), influenced by the business context and its environmental factors. The shift in leadership theory of late is to the context of leadership and what the situation at hand requires, as opposed to in the past, the traits and personalities of individuals. Leadership in a time of constant change and complexity is about three things: *context, context*, and *context*.

### 2.2 Context as a General Principle

In the International Leadership Association's (ILA) General Principles for Leadership Programs (2021) document, *context* is depicted *as a world of rapid change and critical, seemingly intractable local and global problems*. Leadership programs, it continues, foster optimism and the desire to bring about positive and transformative change. They prepare learners to be agile, open‐minded, and humble. Learners deal with increasing volatility, uncertainty, complexity, and ambiguity. The best leadership programs encourage a global, as well as local, perspective and a respectful, holistic systems approach to engaging multiple stakeholders (ILA 2021).

The key questions and issues addressed in this section of ILA General Principles are: How does the context of leadership learning affect the program? and why is it important to consider context for leadership learning? For the ILA, the context for leadership learning is informed by global, national, local, and organizational factors. It also incorporates history, traditions, culture, and language. Of equal importance is the individual's contribution to this context, via their identity, background, values, knowledge, and experience. Leadership learning, in this instance, both promotes a global and a local outlook in terms of knowledge development and impact, while also responding to the tensions that exist between global and local forces, simultaneously recognizing how leadership must endeavor to reconcile these.

In the ILA General Principles document (2021), the subject of context is then explored at the global, regional, national, and organizational levels, in setting the scene, so to speak, for leadership and how to embed this important component in leadership programs. What follows is a rumination on the leadership journey and lessons learnt by the authors on the experiences of the revitalization and implementation of the Executive MBA program at the African Leadership University School of Business (ALUSB), which has embedded the ILA General Principles in its design pedagogy and delivery methodology. The Leadership Lab, which is a core course in the Executive MBA at the African Leadership University (ALU), is informed by and has embedded the International Leadership Association's General Principles for Leadership Programs (2021) that fall within each of the five topical areas in this document—*context, conceptual framework, content, learning, and metrics*.

## 3 Overview of the African Leadership University

The African Leadership University (ALU) was established to transform higher education across the African continent by producing graduates equipped to address Africa's unique challenges. Founded in 2015 by educational entrepreneur Fred Swaniker, ALU has campuses in Mauritius and Rwanda and aims to develop ethical and entrepreneurial leaders who will drive economic growth and social transformation throughout Africa. Unlike traditional universities, ALU offers a mission‐driven curriculum that emphasizes experiential learning, leadership development, and critical thinking over rote memorization. Students at ALU declare leadership as a ‘mission’ rather than a ‘major,’ aligning their education with personal goals and the continent's broader needs, fostering a sense of responsibility and purpose in each student's learning journey.

The university's unique pedagogy is structured around ‘meta‐skills’ essential for success in the 21st century, such as problem‐solving, adaptability, and quantitative reasoning. The ALU aims to revolutionize higher education in Africa by producing ethically grounded leaders, fostering sustainable development, and promoting innovation that aligns with Africa's unique needs. Critical to the achievement of these goals is the leadership context in terms of relevance and responsiveness to the needs of the African political, economic, social, and human development.

**FIGURE 1:**
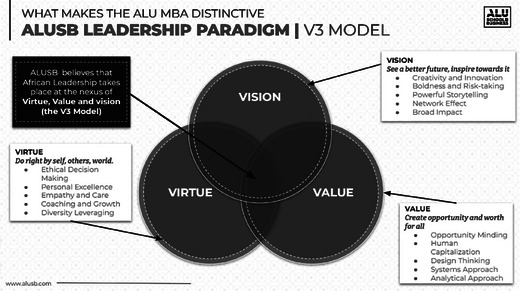
ALUSB V3 model.

The Leadership Lab is grounded in the ALU V3 Leadership Model (see Figure 1), which focuses on Virtue, Value, and Vision (ALU 2016).

Virtue at its core is doing right by self, others, and the world—it means being an ethical actor who uplifts and develops others. This is *who a leader is* and advances the African philosophy of *Ubuntu*. Although critical, this lands softly if a leader does not create opportunity and worth for all involved. In short, Value which we might understand as *what a leader does* or contributes to an organization. In tandem, Virtue and Value make ethical and effective leaders worth joining; yet one thing remains outstanding according to the ALU V3 model. Vision. Vision is *how leaders achieve* their hoped‐for reality, their approach to the bigger picture or dream, and how to achieve it. Leaders cast an imagined view of the future, seeing a bett*er* world and inspiring everyone to reach through their *vision*.

One of the summative individual assignments the EMBA students had to complete and submit in Leadership Lab Module I was a case study, on their personal leadership journey, profiling themselves as a V3 leader, highlighting how their leadership embodies Virtue, Value, and Vision.

## 4 Leadership Is About Context, Context, and Context

To provide the reader with the contextual specifics of the leadership journeys of the MBA student cohort, the authors have selected two case study submissions as a purposive sample, which, according to Palinkas et al. (2015), is widely used in qualitative research for the identification and selection of information‐rich cases related to the phenomenon of interest. In this case, the contextual realities and specificities of the leadership context. Seale (2019) described ‘leadership context’ as encompassing four elements—global, local (or national), institutional/organizational, and individual. There is a complex and changing leadership context in the contemporary setting for the MBA leaders, characterized by global, national, and organizational imperatives or drivers that influence and impact leadership.

Of equal importance is what the individual brings to this context in terms of their background, knowledge, and experience. For instance, their values, beliefs, culture, and role models. The contextual setting for the young African leaders participating in the ALU MBA finds expression through the global and local challenges they are experiencing, leadership and management molding in their respective organizations, and their theoretical reconceptualization and practical application of lessons being learned, both individually and collectively with their peers. To respect and protect their identity and ensure anonymity, we will, in this paper, call our students ‘Joe’ and ‘Mary.’ They are leaders in the areas of environmental services and entrepreneurship.

### 4.1 Joe's Story

Throughout his career in conservation, Joe has been navigating and addressing the complex challenges in Kenya's conservation efforts, such as climate change, habitat degradation, forest depletion, tourism market volatility, human‐wildlife conflict, and wildlife crime. These issues, says Joe, are exacerbated by the high youth unemployment rate, with over one million young people entering the labor market annually without sufficient skills. At a personal level, the loss of his father at an incredibly young age and its impact on his upbringing, the economic challenges associated with single‐parenthood, and the opportunity and support from a university scholarship, for Joe, ‘ignited his passion for youth leadership, mentorship, and social entrepreneurship’.

In terms of embracing and advancing the ALU V3 leadership model, Joe's visionary approach involves acting boldly and engaging others through powerful narratives. He has built a constituency of conservationists through his work and now as a venture builder. By tapping into the power of networks, Joe has started ventures that create positive impacts, such as job creation and poverty reduction. His work in conservation has demonstrated his ability to envision a better future and take the necessary steps to realize it. Joe's leadership journey, driven by a desire to give back and empower others, continues to inspire young people to emulate his path. His unwavering commitment to ethical leadership, market‐driven innovation, and bold visionary goals serves as a beacon for future generations of leaders. So, for Joe, his professional and leadership journey, as related in his leadership story, has been influenced by these contextual realities and is a testament to overcoming the local systemic challenges through innovative solutions and impactful leadership.

### 4.2 Mary's Story

Following Sani Abacha's repressive military government rule in Nigeria (1993–1998) and the recovery of its economy, opportunities were created for Mary's parents to set up a small enterprise. This experience in a changing context provided her with personal insight into the challenges faced by Nigerian entrepreneurs with limited resources at that time. With this background, Mary pursued her tertiary education in Management Information Systems, wherein she encountered a liberal arts academic experience that also focused on servant leadership. Her interest in business grew during her time at university, particularly as she observed a recurring trend in her studies: the dominance of multinational corporations over African‐owned companies, constituting only 2% of successful enterprises at that time. This observation led Mary to focus her final year thesis title on, *How African‐owned businesses could thrive in competition with multinational corporations*.

For Mary, the desire for and commitment to supporting local businesses from their inception, through their start‐up phase, onto a growth trajectory, while balancing this with the demands of a young mother, became her lived reality. Initially opting for roles with significant remote work flexibility, Mary endeavored to contribute to the support of African businesses while still managing her personal responsibilities at home. However, she soon realized that remote work limited her ability to understand and address the challenges these businesses faced on a day‐to‐day basis. She recognized that achieving impactful change in a business required a leader's physical presence, availability, and personal connection with their team. The feedback she was receiving in this regard confirmed this. At this point, Mary faced a typical work‐life balance issue, leading to her own reflection on: *How can she develop herself and her career while prioritizing her family?*

Around this time, Mary registered for the ALU's MBA program, which introduced her to the V3 Leadership model and immersed her in various case studies of leaders across the African continent who had made impactful changes. One case that particularly challenged Mary in her Leadership Lab module was that of Dr. Hawa Abedi, a leader who significantly impacted her generation despite being a wife and mother (DHAF 2025). Hawa Abdi was a remarkable Somali human rights activist and physician. Born on May 17, 1947, in Mogadishu, she became Somalia's first female gynecologist after studying medicine in Kiev. She later earned a law degree from the Somali National University. In 1983, she founded the Rural Health Development Organization (RHDO), which evolved into the Dr. Hawa Abdi Foundation (DHAF). Her foundation provided free healthcare, education, and shelter to thousands of displaced Somalis, especially women and children. Dr. Abdi's work earned her numerous awards and nominations, including a Nobel Peace Prize nomination in 2012. She passed away on August 5, 2020, but her legacy continues through her daughters and the foundation (DHAF 2025)

This case study resonated with Mary in terms of her own struggle with commitment to African businesses as well as the nurturing needs of her young family. For Mary, the main challenge was that she was in a comfort zone and needed a change of mindset to find and maintain the balancing act between professional and personal roles and commitments. The key lesson for Mary was that, given Hawa Abedi's leadership story, she could help many African people and businesses more in‐depth, inspired also by her father's business story and the companies she had interacted with. She could still find ways to help develop people in rural areas with different lifestyles, limited resources, and perspectives. And simultaneously, be a caring and present mother. Mary's contextual realities were not a case of professional versus personal conflict but more about the drive and commitment to follow her professional fervor while being grounded in her role as a mother.

## 5 Lessons and Implications

The ALU MBA class of 2024 comprises 22 students from eight African countries, which are Kenya, Benin, Rwanda, Mauritius, South Africa, Zimbabwe, Nigeria, and Tanzania, from various sectors including Information Communication and Technology, Environmental Services, Consulting, Government and International Organizations, Education, Media, and Non‐profit Organizations. Their average age is 35 years. This context is key to their leadership stories.

Storytelling is a powerful tool in leadership. It can inspire, motivate, and connect people on a deeper and more meaningful level. The personal leadership stories of the MBA students were grounded in, and demonstrated, the role of context in a leader's journey and how it influences their thinking and actions. What the authors observed was that the shared experience of diverse backgrounds and personal histories provided not only a rich repository of knowledge but also contributed to a deeper understanding of the individual and group dynamics.

The class engagements on the contextual realities of leadership were robust and dynamic, and took place in an enabling and empowering environment. From the students’ assignments, we have seen their leadership growth path and maturity as professionals in a leadership learning context of vulnerability, trust, and support for themselves and one another. The leadership learning pedagogy as advanced by the ILA General Principles for Leadership Programs (2021) framework and the ALU applied learning methodology has for the MBA leaders, created a platform and vehicle for personal and professional discovery, and the acquisition of knowledge and skills in their own organizational environments, cognizant of their contextual space, and its implications for their own leadership journeys.

## 6 Conclusion

For Warren Bennis (1989), *Leadership is like beauty, it's hard to define but you know it when you see it!* This portrayal of leadership demonstrates what leaders do and is more important than what they say, or simply put, actions speak louder than words. The founding father of democracy in South Africa, Nelson Mandela (2011) said, “A leader…is like a shepherd. He stays behind the flock, letting the nimblest go out ahead, whereupon the others follow, not realizing that all along they are being directed from behind.” This philosophy is at the heart of African leadership and the ubuntu philosophy, with its focus on the collective, not the individual. The applied learning methodology of the ALU MBA grounded in the theoretical framing of *context* in leadership learning, has shown that the background, experiences, and perspectives form the foundation of the students’ leadership journey and evolving stories. There is strength in diversity and the composition and demographics of the ALU MBA leadership class speaks to the criticality of understanding identity, self‐awareness, and connectedness to the local, national, and regional leadership context. What we have seen and continue to experience and are advancing at ALU, is that the conceptual framing and applied reality of leadership, is about context, context, and context! Moreover, the need for further research and the pursuit of an African leadership education and development road map that encompasses V3 Leadership but is also grounded in the realities of a complex, changing environment, should be theorized and implemented for praxis.
